# A PILOT AND FEASIBILITY STUDY OF THE AGING TOGETHER ANTI-AGEISM PEER SUPPORT PROGRAM

**DOI:** 10.1093/geroni/igae098.4326

**Published:** 2024-12-31

**Authors:** Andrew Steward, Connor Thomas Francisco Keane, Yura Lee, Young Cho

**Affiliations:** University of Wisconsin-Milwaukee, Milwaukee, Wisconsin, United States; University of Wisconsin-Milwaukee, Milwaukee, Wisconsin, United States; University of Wisconsin-Milwaukee, Milwaukee, Wisconsin, United States; University of Wisconsin-Milwaukee, Milwaukee, Wisconsin, United States

## Abstract

Ageism is internalized from an early age, with accumulating, negative health impacts across the life course. Although the theory of relational ageism describes how ageism is reinforced through interpersonal interactions, few ageism-related intervention studies have actively engaged older adults at personal and relational levels. We conducted a pilot and feasibility study of a manualized, anti-ageism peer support program facilitated by trained older adults called “Aging Together.” Key components of the program include peer support, education about ageism and health, and “tell your story of aging.” Participants received a 30-page workbook providing education and journaling/discussion prompts. Six small groups of five-to-eight participants (N=48 total) met for ten, weekly, in-person (60-90 minute) sessions in a medium-sized city of the U.S. Midwest. Results indicate the program was feasible and acceptable overall (attendance rate=88.91%, attrition rate=6.25%, Mean acceptability score=4.23/5). Based on 30-minute pre/post phone surveys, Mann-Whitney U tests (N=28) indicated a significant decrease in relational ageism (M=2.73/6 to 2.19/6; p<.01), decrease in internalized ageism (M=1.91/6 to 1.66/6), significant decrease in depressive symptoms (M=8.61/30 to 6.57/30; p<=.01), increase in social connectedness (M=4.19/5 to 4.26/5), increase in self-efficacy (M=3.29/4 to 3.36/4), and decrease in cognitive function (M=29.67/40 to 29.5/40). By incorporating education about ageism and storytelling within a small, peer support group setting, the Aging Together program may be particularly effective in reducing depressive symptoms and relational ageism, which is an understudied aspect of ageism. Future research should scale this program across diverse geographical settings and explore potential mediating relationships between internalized/relational ageism and psychosocial well-being.

